# Characterisation of *Vibrio* Species from Surface and Drinking Water Sources and Assessment of Biocontrol Potentials of Their Bacteriophages

**DOI:** 10.1155/2020/8863370

**Published:** 2020-08-04

**Authors:** Mpho Defney Maje, Christ Donald Kaptchouang Tchatchouang, Madira Coutlyne Manganyi, Justine Fri, Collins Njie Ateba

**Affiliations:** ^1^Food Security and Safety Niche Area, Faculty of Natural and Agricultural Sciences, North-West University, Mmabatho, Mafikeng 2735, South Africa; ^2^Department of Microbiology, School of Biological Sciences, Faculty of Natural and Agricultural Sciences, North-West University, Private Bag X2046, Mmabatho, South Africa

## Abstract

The aim of this study was to characterise *Vibrio* species of water samples collected from taps, boreholes, and dams in the North West province, South Africa, and assess biocontrol potentials of their bacteriophages. Fifty-seven putative *Vibrio* isolates were obtained on thiosulfate-citrate-bile-salt-sucrose agar and identified using biochemical tests and species-specific PCRs. Isolates were further characterised based on the presence of virulence factors, susceptibility to eleven antibiotics, and biofilm formation potentials. Twenty-two (38.60%) isolates were confirmed as *Vibrio* species, comprising *V. harveyi* (45.5%, *n* = 10), *V. parahaemolyticus* (22.7%, *n* = 5), *V. cholerae* (13.6%, *n* = 3), *V. mimicus* (9.1%, *n* = 2), and *V. vulnificus* (9.1%, *n* = 2). Three of the six virulent genes screened were positively amplified; four *V. parahaemolyticus* possessed the *tdh* (18.18%) and *trh* (18.18%) genes, while the *zot* gene was harboured by 3 V. *cholerae* (13.64%) and one *V. mimicus* (4.55%) isolate. Isolates revealed high levels of resistance to cephalothin (95.45%), ampicillin (77.27%), and streptomycin (40.91%), while lower resistances (4.55%–27.27%) were recorded for other antimicrobials. Sixteen (72.7%) isolates displayed multiple antibiotic-resistant properties. Cluster analysis of antibiotic resistance revealed a closer relationship between *Vibrio* isolates from different sampling sites. The *Vibrio* species displayed biofilm formation potentials at 37°C (63.6, *n* = 14), 35°C (50%, *n* = 11), and 25°C (36.4%, *n* = 8). Two phages isolated in this study (vB_VpM_SA3V and vB_VcM_SA3V) were classified as belonging to the family Myoviridae based on electron microscopy. These were able to lyse multidrug-resistant *V. parahaemolyticus* and *V. cholerae* strains. These findings not only indicate the presence of antibiotic-resistant virulent *Vibrio* species from dam, borehole, and tap water samples that could pose a health risk to humans who either come in contact with or consume water but also present these lytic phages as alternative agents that can be exploited for biological control of these pathogenic strains.

## 1. Introduction

Infections caused by pathogenic *Vibrios* remain a severe threat to the public. Most of these infections result from the consumption of undercooked seafood products or contaminated water [1]. Also, person-to-person transmission has been documented [2]. These infections are classified into cholera and noncholera types [3]. *Vibrio cholerae* infections can be fatal if not properly managed [4, 5]. Noncholera infections range from self-limiting gastroenteritis to severe life-threatening septicaemia and necrotizing fasciitis [1]. *V. cholerae* and *V. parahaemolyticus* are mostly associated with human infections [6, 7]. However, other *Vibrios*, such as *V. alginolyticus*, *V. harveyi*, *V. anguillarum*, *V. mimicus*, *V. metschnikovii*, *V. vulnificus*, and *V. fluvialis*, which have been detected, particularly in marine environments, are now considered as emerging human pathogens [8].

A variety of virulence factors are exhibited by pathogenic *Vibrios* responsible for cholera and noncholera infections. Generally, the ability of *Vibrio cholerae* to cause disease largely depends on the production of the toxin-coregulated pilus (TCP) and the cholera toxin (CT). The integrated prophage CTX*ϕ*-located *ctxB* and *ctxA* genes encode the CT, which is implicated in diarrhoea with dehydration and electrolyte loss [9]. In contrast, TCP coded by *tcpA* aids the pathogen to colonise the epithelium of the small intestine. The *ToxR* regulon regulates the expression of these genes in response to external stimuli [9]. The major contributors to *V. parahaemolyticus* pathogenicity are the thermostable direct haemolysin (*trh*) and the thermostable direct haemolysin-related (*trh*) genes [10–12]. The biological impacts of these proteins include hemolytic as well as cytotoxic effects [13]. The virulence of *V. vulnificus* is encoded by the *vcg* gene. The degree of virulence in this species is related to its origin whereby higher virulence is demonstrated by clinical isolates than environmental strains. Conversely, *Vibrio fluvialis* has been shown to produce numerous potent toxins. Just as in *V. cholerae*, the *ToxR* gene is an important virulence determinant of *V. fluvialis*. Others include the heat stable enterotoxin*, hupO*, *vfp*, and *vfh* genes [14, 15].

Antibiotics and other antimicrobial agents have been used, since their discovery, for the treatment and management of bacterial infections in humans and animals [16]. The treatment of *Vibrio* infections in humans includes the use of doxycycline as the preferred drug for the elderly, while azithromycin is recommended for pregnant women and children [1]. Unfortunately, there is an ever-increasing resistance displayed by several bacterial strains against commonly used and recommended antimicrobial agents. Antibiotic resistance, therefore, is a severe challenge to therapy and can account for a large proportion of therapeutic failures, resulting in high morbidity and mortality [17, 18]. Consequently, immediate solutions are needed to limit the spread of resistant bacteria/determinants.

The biofilm-producing ability of bacterial species further complicates antibiotic resistance. Such strains are enclosed in an exopolysaccharide matrix component adhered to a solid surface, which provides both structural and protective functions to bacterial strains. The ability to form biofilms is, therefore, a fundamental component to ensure environmental survival and transmission [19, 20]. Properties expressed by biofilm-forming cells are distinct from those of planktonic cells. One of such properties is increased resistance to antimicrobial agents compared to planktonic cells [19]. Bacteria biofilms, even when present at very low detection limits in water, act as a constant environmental reservoir for continual water contamination [21].

With the increase in antibiotic-resistant human pathogenic infections, there is a renewed interest in the search for novel and alternative therapeutic or biocontrol agents against clinically relevant bacteria. Bacteriophages are regarded as promising bacterial agents, highlighting their importance as novel therapeutic agents [22, 23]. Some studies have revealed that the combination of different phage stocks in a single experiment improves the host range. Such combination also enhances virulence capabilities against resistant strains compared to individual phages [24, 25].

Consumption of contaminated water is one of the sources of *Vibrio* infections. South Africa is located in a semiarid region and receives very less rainfall, resulting in shortage of potable water. Most individuals, therefore, resort to water from unprotected sources, such as rivers, boreholes, and dams, for daily activities, such as irrigation, cooking, and even drinking [26]. Unfortunately, microbial contamination of such unprotected water bodies is a significant cause of a large proportion of water-associated epidemics [27–29]. The significant number of reports confirming the presence of pathogenic microorganisms in water consumed by people in rural communities in the North West province of South Africa also informed the need to conduct the study [30–34]. Thus, in this study, virulent *Vibrio* species of water samples collected from taps, boreholes, and dams in the North West Province, South Africa, were characterised, and the lytic potentials of *Vibrio*-specific bacteriophages were assessed for potential exploitation of biocontrol agents against pathogenic *Vibrio* strains.

## 2. Materials and Methods

### 2.1. Ethical Clearance

This study was approved by the Research Ethics Committee of the North-West University, South Africa (ethical clearance number: NWU-00725-18-A9).

### 2.2. Collection of Samples

**Figure 1:**
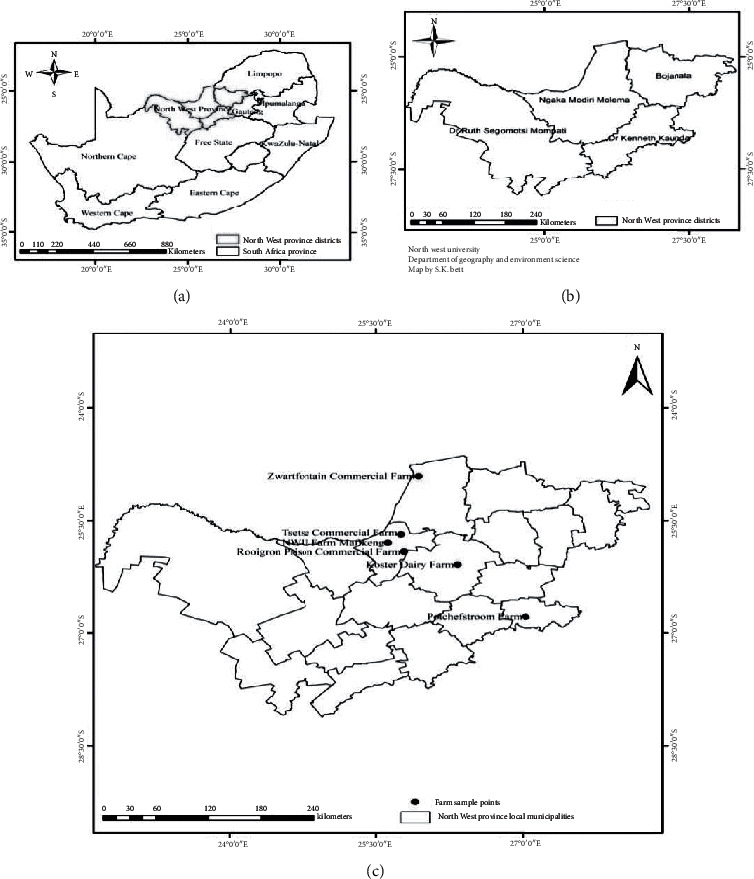
Sampling sites.

One hundred and thirty-six (136) water samples were collected from taps, boreholes, and dams from 20 randomly selected areas and communities in the North West province, South Africa (Figure 1) using sterile 500 mL Duran Schott bottles. For borehole and tap water samples, taps were allowed to run for about five minutes to purge water from the pipes and draw fresh water from the water supply system. The lid from the sample container was removed, and without touching the inside of the bottle or lid, the container was filled with 100 mL of water. For dam water samples, the lid from the sample container was also removed, and without touching the inside of the bottle or lid, the container was immediately filled with 100 mL of water. The lid was tightly closed to prevent leakage. The samples were properly labelled and transported on ice to the laboratory for analysis. The number of water samples collected from different areas is presented in Supplementary Table 1. These sites were selected because previous studies in the North West province have revealed the presence of virulent and multidrug-resistant strains in either food or water samples [30–34]. The choice of these sites not only reflects the desire to contribute towards finding potential solutions regarding the worrying trends associated with challenges of treating infections resulting from the spread of resistant bacterial strains within communities but also in addressing problems that are common in our immediate local environment.

### 2.3. Sample Processing and Isolation of *Vibrio* Species

Water samples were analysed immediately upon arrival in the laboratory. For each water sample, 100 mL was filtered through 0.45 *μ*m membrane filters (Sigma-Aldrich, Missouri, USA), and the filter papers were inoculated on thiosulfate-citrate–bile-salt-sucrose (TCBS) agar (Merck, Darmstadt, Germany) [35]. Plates were incubated aerobically at 37°C for 24 hours. After incubation, colonies with different morphotypes were subcultured on TCBS, and plates were incubated aerobically at 37°C for 24 hours. Pure isolates were stored at 4°C for further analysis. Preliminary identification of isolates was done using Gram staining, biochemical tests (oxidase test, triple sugar iron agar, and Simmons citrate agar), salt tolerance, and motility tests. Presumptive isolates were stored in 20% (v/v) glycerol at −80°C for future analysis.

### 2.4. Molecular Characterisation of *Vibrio* Species

Genomic DNA was isolated from all presumptive *Vibrio* isolates using the cetyltrimethylammonium bromide (CTAB) method with slight modifications [36]. The quality and purity of the DNA were determined using a spectrophotometer (version UV-visible spectrophotometer model S-22, Boeco, Germany) at wavelengths of 260 nm and 280 nm. Molecular identification of isolates was performed using polymerase chain reaction (PCR) assay. Fragments of the bacterial 16S rRNA gene were amplified as an internal control for all presumptive isolates, using universal oligonucleotide primers 27F and 1492R [37], while species-specific primers were used to identify species. Target genes included *sodB* (1) and *ompW* for *V. cholerae, rfb* specific for *V. cholerae* serogroup O1, the *flaE* gene for *V. parahaemolyticus*, *hsp* for *V. vulnificus*, *sodB* for *V. mimicus*, and *vhh* for *V. harveyi* (Table 1).

### 2.5. Detection of *Vibrio* Virulence Genes

Virulence gene determinants in *Vibrio* species were determined by PCR amplification of the *tdh, trh, ctxAB, zot, flrA*, and *vpsR* gene sequences. These genes, which were thought to only be specific to either *V. parahaemolyticus* (*tdh* and *trh*) or *V. cholerae* (*ctxAB*, *zot*, *flrA*, and *vpsR*), have been detected from other environmental *Vibrios* [8]. The oligonucleotide sequences, target genes, amplicon sizes, and cycling conditions of the PCR assays are listed in Table 2. All PCR reactions were prepared in a standard 25 *μ*L volume, comprising 12.5 *μ*L of 2X DreamTaq Green Master Mix (0.4 mM dATP, 0.4 mM dCTP, 0.4 mM dGTP, and 0.4 mM dTTP, 4 mM MgCl_2_, and loading buffer) (Fermentas, USA), 11 *μ*L nuclease-free water (Fermentas, USA), 0.25 *μ*L of each forward and reverse primers (Inqaba Biotechnologies, Pretoria), and 1 *μ*L of template DNA. Amplifications were performed in a C1000 Touch™ Thermal Cycler (Bio-Rad, UK). Amplicons were resolved by electrophoresis on a 2% (w/v) agarose gel at 90 V for 50 minutes and visualised using a ChemiDoc™ MP Imaging System (Bio-Rad, UK).

### 2.6. Antimicrobial Susceptibility Test

The Kirby–Bauer disc diffusion assay was used to determine the antimicrobial resistance patterns of confirmed *Vibrio* isolates in accordance with the guidelines of the Clinical Laboratory Standards Institute [42, 43]. *Vibrio* isolates were screened against a panel of eleven antimicrobial agents (Mast Diagnostics, UK) belonging to seven classes. A bacterial suspension of each pure isolate was prepared in 0.8% (w/v) sterile physiological saline, vortexed, and the optical density adjusted to the 0.5 McFarland standards. Aliquots of 100 *μ*L inoculum from each suspension were spread-plated onto Mueller–Hinton agar plates. Antimicrobial-impregnated discs were placed at equal distances on the inoculated plates. The antimicrobial agents included the following: ampicillin (AMP), 10 *μ*g; cephalothin (CEF), 30 *μ*g; chloramphenicol (CHL), 30 *μ*g; ciprofloxacin (CIP), 5 *μ*g; tetracycline (TET), 30 *μ*g; gentamicin (GEN), 10 *μ*g; kanamycin (KAN), 30 *μ*g; nalidixic acid (NAL), 30 *μ*g; trimethoprim-sulfamethoxazole (TS), 1.25/23.75 *μ*g; streptomycin (STR), 10 *μ*g; and trimethoprim (TMP), 5 *μ*g. The plates were incubated at 37°C for 24 hours, and the inhibition zone diameters were measured. Values obtained were interpreted in accordance with the CLSI interpretive criteria [43].

### 2.7. Biofilm Formation Assay

Biofilm formation assay was performed in a 96-well microtiter plate according to the method described earlier [44]. In brief, bacteria were grown in nutrient broth (NB) at 37°C for 24 hours. Two hundred microlitres of 1 : 100 overnight cultures in fresh NB were dispensed into wells and incubated at 37°C for 24 hours. Negative control wells consisted of uninoculated sterile NB. The contents of wells were discarded and washed twice with phosphate buffer saline (PBS). Two hundred microlitres of 1% (w/v) crystal violet dye were added to each well followed by incubation of plates at room temperature for 1 hour. The dye was discarded, and the wells were washed five times with PBS and allowed to dry at room temperature. Two hundred microlitres of 95% (v/v) ethanol were added to the wells, with further incubation at room temperature for 5 minutes. Ethanol was transferred to wells of new microtiter plates, and the optical density at 630 nm was recorded. The protocol was repeated with initial bacterial incubation of the plates at 35°C and 25°C. The biofilm-producing potential of isolates was classified as follows: biofilm formation if OD_test_ < OD_control_; weak biofilm formation if OD_control_ < OD_test_ < 2OD_control_; moderate biofilm formation if 2OD_control_ < OD_test_ < 4OD_control_; and strong biofilm formation if 4OD_control_ < OD_test._

### 2.8. Bacteriophage Assay

#### 2.8.1. Isolation of *Vibrio* Phages

Isolation of phages was done using the double agar layer method [45]. Five of the PCR-confirmed *Vibrio* isolates were used to isolate bacteriophages from sewage water. Ten millilitres of water were suspended in 10 mL of lambda diluent (10 mM Tris HCl (pH 7.5) and 8 mM MgSO_4_.7H_2_O), vortexed thoroughly, and incubated at room temperature for 60 minutes. This was followed by centrifugation of the samples at 5,250 ×g for 10 minutes to sediment the bacterial cells. Each supernatant was extracted and further centrifuged followed by filtering through a 0.22 *μ*m pore syringe filter (GVS Filter Technology, USA). Crude filtrates were enriched in Tryptic Soy Broth (TSB) and incubated at 37°C for 60 minutes.

Phage activity was determined using the following procedures: ten-fold serial dilutions of the phage lysates were prepared using lambda diluent; aliquots (100 *μ*L) of each dilution were added to 100 *μ*L of log phase *Vibrio* species host bacteria cultured in TSB containing 10 Mmol/L MgSO_4_; the mixture was incubated at room temperature for 10 minutes to facilitate attachment; an aliquot of 3 mL of 0.6% (w/v) agarose, cooled to about 50°C, was added into each mixture, and the contents were poured on solidified modified nutrient agar (MNA); and plates were incubated aerobically at 37°C for 24 hours followed by observation of the plaques.

#### 2.8.2. Purification, Enumeration, and Propagation of Phages

Plaques were picked from plates using sterile pipette tips and inoculated in 1.5 mL of Difco phage broth (DPB) and incubated overnight at 4°C for phages to diffuse out of agarose. Phages in the lysates were purified three times in succession by single-plaque isolation, and in each case, stock filtrates were prepared using suitable host strains as described previously [46]. Titers of isolated phages in stock filtrates were also determined using the soft layer overlay technique [47]. The purified bacteriophage lysates were stored at 4°C.

Phage propagation was carried out by centrifuging phages in DFB at 10,000 ×g for 2 minutes, and an equal volume (1 mL) of the resulting lysate and overnight broth culture of each *Vibrio* strain were added to 5 mL of DPB. The mixture was incubated at 37°C in a shaking water bath for 6-7 hours. The phage lysates were centrifuged at 9,000 ×g for 15 minutes to remove bacterial debris, and the supernatant was filtered through a 0.2 *μ*m pore-size Super Acrodisc syringe filter.

### 2.9. Characterisation of Bacteriophages

#### 2.9.1. Electron Microscopy

Phages were purified using polyethylene glycol (PEG), in accordance with standard procedures [48]. Phage suspensions were centrifuged at 25,000 ×g for 60 minutes, and the resulting pellets were washed twice in ammonium acetate (0.1 mol l^−1^, pH 7.0). Pellets were subjected to transmission electron microscopy. Each phage was assigned a name as previously described [49], comprising vB (bacterial virus) followed by Vp and (*V. parahaemolyticus*) or Vc (*V. cholerae*), M (*Myoviridae*), SA (South Africa), numbers (sample identity) followed by K or V, etc., (sampling site). For example, a phage isolated from Ventersdorp using *V. parahaemolyticus*, as the host strain, was designated vB_VpM_SA3V.

#### 2.9.2. Microplate Phage Virulence Assay and Host Specificity

The lytic capabilities of the phages were assessed using the microplate phage virulence assay as earlier described [46] with slight modifications. Aliquots of 180 *μ*L of each of the 22 overnight bacteria cultures were mixed with 20 *μ*L of filtered phage lysates in a 96-well microtiter plate and incubated at 37°C for 5 hours. TSB was used as a blank, while the overnight cultures were used as negative controls. Treatments were carried out in triplicates. Wells were examined visually for turbidity due to bacterial growth. Also, a spectrophotometer was used to read the results and compare the OD readings with results from the visual analysis of the plates. Results were interpreted as positive when there was inhibition of bacterial growth or negative when bacterial growth was not inhibited.

#### 2.9.3. Stability of Varying Temperatures and pH of Phages

The stability of the phages at different pH and temperatures was assessed as previously described, with slight modifications [46]. For the stability of phages to various pH, 100 *μ*l of phage lysates was added to 900 *μ*l of pH-adjusted TSB (0, 4.2, 6.3, 7, 8, and 10). The tubes were incubated at room temperature for 5 hours followed by the determination of phage titer using the soft agar overlay technique. The stability of phages to different temperatures was determined by incubating phage lysates of known concentrations at 37°C, 45°C, 55°C, and 60°C for 1 hour followed by the determination of phage titers using the soft agar overlay method.

### 2.10. Statistical Analysis

Bacteria growth inhibition zone diameter data of isolates from different stations were used to perform a cluster analysis, using the Wards algorithm and Euclidean distances on Statistica software, version 12 (Statsoft, US). The analysis was done to determine the relatedness of isolates based on the history of antibiotic exposure.

## 3. Results

### 3.1. Occurrence of *Vibrio* in Water Samples

Fifty-seven nonrepetitive presumptive *Vibrio* isolates that presented different macroscopic colonial morphologies were obtained through the culture. Out of this number, 20 were from tap water, 32 from boreholes, and 5 from dam water. All the isolates were Gram-negative rod-shaped bacteria that fermented the sugars in the TSI medium and did not result in blackening of the medium, thus unable to produce hydrogen sulphide gas. The isolates were unable to utilise sodium citrate as the sole source of carbon. All the isolates grew at 0%, 3%, 6%, and 10% (w/v) NaCl solution, with the exception of one isolate that did not tolerate 6% and 10% salt.

**Figure 2:**
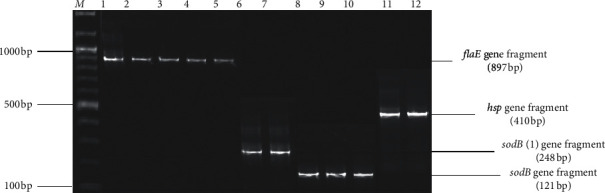
A 2% (w/v) agarose gel, showing amplicons of fragments of *flaE* (*V. parahaemolyticus*), *hsp 60* (*V. vulnificus*), *sodB* (1) (*V. cholerae*), and *sodB* (*V. mimicus*) genes amplified during the study. Lane *M* = 100 bp DNA marker, lanes 1–5 = fragments of the *flaE* gene of positive isolates of *V. parahaemolyticus*, lanes 6-7 = fragments of the *sodB* (1) gene amplified from positive isolates of *V. cholerae*, lanes 8–10 = fragments of the *sodB* gene of positive isolates of *V. mimicus*, and lanes 11-12 = fragments of the *hsp 60* gene of positive isolates of *V. vulnificus*.

**Figure 3:**
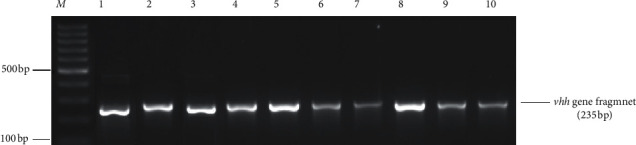
A 2% (w/v) image of agarose gel showing fragments of the *vhh* gene amplified from isolates of *V. harveyi*, lane *M* = 100 bp DNA ladder, and lanes 1–12 = fragments of the *vhh* gene.

Fragments of the bacterial 16S rRNA gene were amplified in all the 57 presumptive isolates and further subjected to *Vibrio* species-specific PCR identification assays. Twenty-two presumptive isolates were identified: a large proportion (45.45% *n* = 10) was detected as *V. harveyi* followed by *V. parahaemolyticus* 5 (22.73%) and *V. cholerae* 3 (13.64%), and the least were *V. mimicus* and *V. vulnificus,* with two (9.1%) isolates each. These constituted 7, 4, and 11 isolates from taps, dams, and boreholes, respectively. A detailed summary of the number of species detected in different samples is given in Table 3, while Figures 2 and 3 are representative gels, showing fragments of different genes amplified for identification of *Vibrio* species.

### 3.2. Prevalence of Virulent Genes

**Figure 4:**
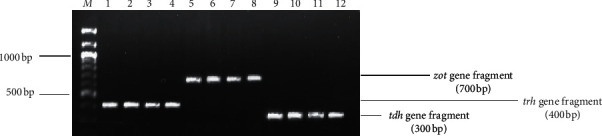
Image of agarose gel showing fragments of virulent genes *zot*, *trh*, and *tdh*. Lane *M* = 100 bp DNA marker, lanes 1–4 = fragments of the *trh* gene, lanes 5–8 = fragments of the *zot* gene, and lanes 9–12 = fragments of the *tdh* gene.

Three of the six virulent genes screened were detected. Four out of five *V. parahaemolyticus* isolates (80%) constituting 18.2% of the total *Vibrios* detected in this study harboured the *tdh* virulent gene. This comprised all three isolates from Lonely Park and one from Ramosadi. Similarly, four *V. parahaemolyticus*, comprising two each from borehole water in Ramosadi and Lonely Park, were positive for *trh*. A similar number, four (18.18%) isolates possessed the *zot* virulent gene, three *V. cholerae* from Pella and one (1) *V. mimicus* from Seweding.  Figure 4 is a 2% (w/v) image of agarose gel obtained from different virulent genes amplified during the study.

### 3.3. Antibiotic Susceptibility Profiles of Isolates

Out of the twenty-two isolates assayed, the highest percentages of resistance were observed for cephalothin (95.5%, *n* = 21) followed by ampicillin (77.3%, *n* = 17), streptomycin (40.9%, *n* = 9), nalidixic acid (27.3%, *n* = 6), and tetracycline (22.7%, *n* = 5). However, 3 (13.6%) isolates were resistant to gentamycin, kanamycin, and trimethoprim, 2 (9.1%) to chloramphenicol, and 1 (4.5%) to trimethoprim-sulfamethoxazole and ciprofloxacin. All isolates displayed resistance properties to at least one antimicrobial tested. Multiple-antibiotic resistance (resistance to 3 or more antibiotic classes) was recorded in 16 (72.7%) isolates with AP^R^-CF^R^-T^R^-S^R^ representing the dominant phenotype observed in 4 (18.2%) isolates.  Table 4 shows the proportion of *Vibrio* isolates that were resistant, intermediately resistant, or susceptible to the antibiotics tested.

### 3.4. Cluster Analysis of *Vibrio* Isolates Based on Inhibition Zone Diameter Data

**Figure 5:**
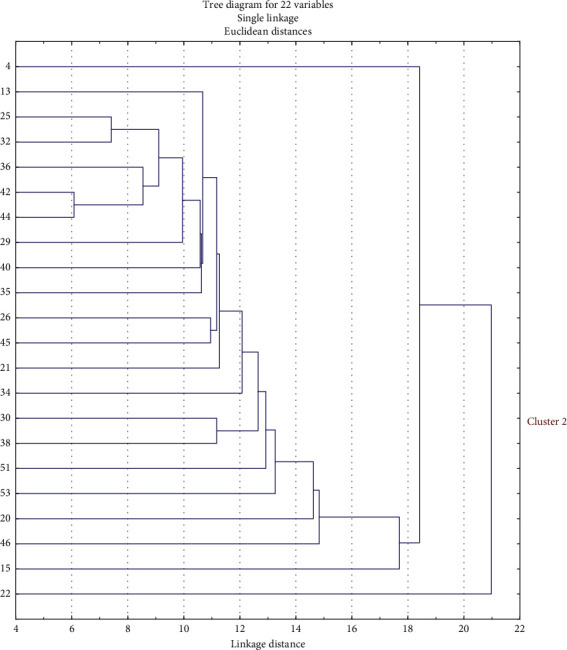
Dendrogram showing the relationship between isolates from water samples based on antimicrobial inhibition zone diameter data.

Cluster analysis was used to determine the relatedness of isolates based on the history of exposure to antibiotics. The inhibition zone diameter data of the twenty-two *Vibrio* isolates to eleven antibiotics were used to perform the analysis, using Statistica software (version 12). A dendrogram comprising two clusters (1 and 2) was generated (Figure 5). Cluster 1 was further subdivided into two subclusters (1A and 1B). Subclusters of 1A, 1B, and cluster 2 were analysed for patterns of association of isolates from different sources and locations. As indicated in Figure 4, the largest subcluster (cluster 1B) contained 20 strains from 10 out of the 11 sampling sites. Cluster 1A constituted the lone isolate from Pudimoe, while cluster 2 included one of the 2 isolates from boreholes obtained from Lonely Park. None of the five isolates obtained from dam water samples was present in either subcluster 1A or cluster 2.

### 3.5. Biofilm Formation Capacity of *Vibrio*

**Figure 6:**
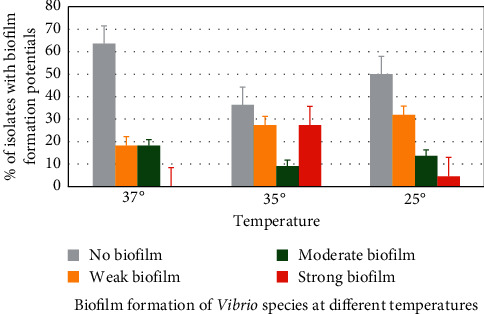
Biofilm formation potentials of *Vibrio* species at different temperatures.

All the twenty-two *Vibrio* isolates were screened in order to determine their potential to form biofilms on polystyrene plates at 37°C, 35°C, and 25°C. Eight (36.4%) isolates formed biofilms (4 moderate and 4 weak) at 37°C, 14 (6 strong, 2 moderate, and 6 weak) at 35°C, and 11 (1 strong, 3 moderate, and 7 weak) at 25°C.  Figure 6 is an overview of the biofilm formation potentials of isolates at different temperatures.

### 3.6. Characterisation of *Vibrio*-Specific Bacteriophages

#### 3.6.1. Transmission Electron Microscopy

**Figure 7:**
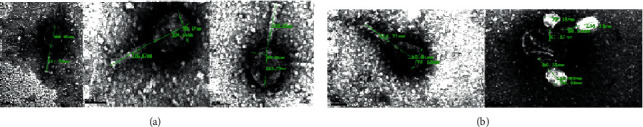
Transmission electron micrographs of *Vibrio*-specific bacteriophages isolated from sewage. (a) vB_VpM_SA3V. (b) vB_VcM_SA3V.

Morphological classification of the phages was achieved using previous guidelines [48]. Both phages had icosahedral heads and long contractile tails (Figure 7). Based on these characteristics, the phages were classified as belonging to the family Myoviridae (Ackermann [48]).

#### 3.6.2. Bacteriophage Virulent Assay

Two bacteriophages designated phage A and phage B, based on their profiles, were used to assess their potentials as biocontrol against all the twenty-two molecularly confirmed environmental *Vibrios* isolated in the study. Results were interpreted as positive when there was inhibition of bacterial growth or negative when bacterial growth was not inhibited. The outcome of virulence assay revealed phage A as biologically active against 19 of the 22 *Vibrio* strains, while phage B was active against 18 isolates. Interestingly, both bacteriophages were active against the same sets of bacterial strains as 17 individual *Vibrio* strains could be inhibited by both phages A and B, two by phage A only, and one by phage B only.

#### 3.6.3. Stability of Phage at Different Conditions

**Figure 8:**
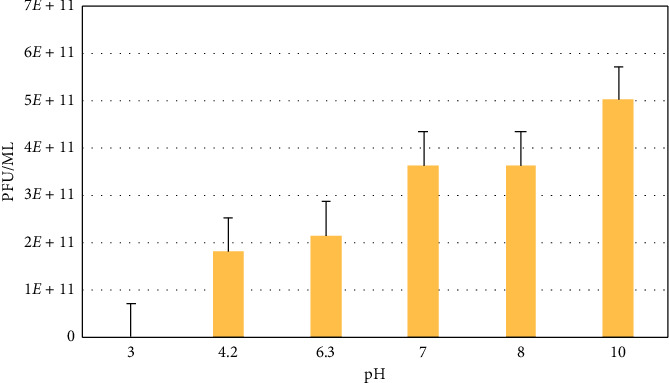
Stability of phage vB_VpM_SA3V at different pH levels.

**Figure 9:**
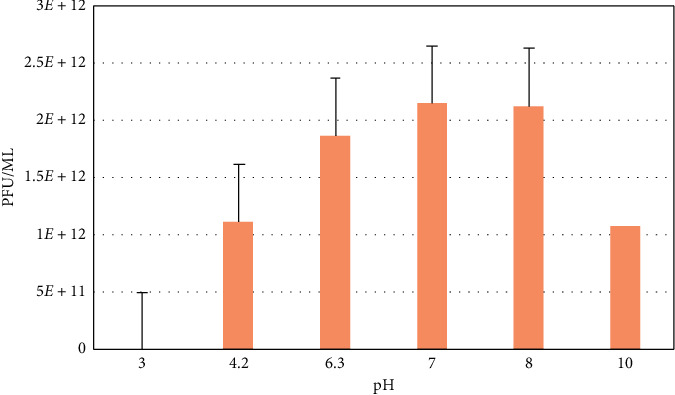
Stability of phage vB_VcM_SA3V at different pH levels.

**Figure 10:**
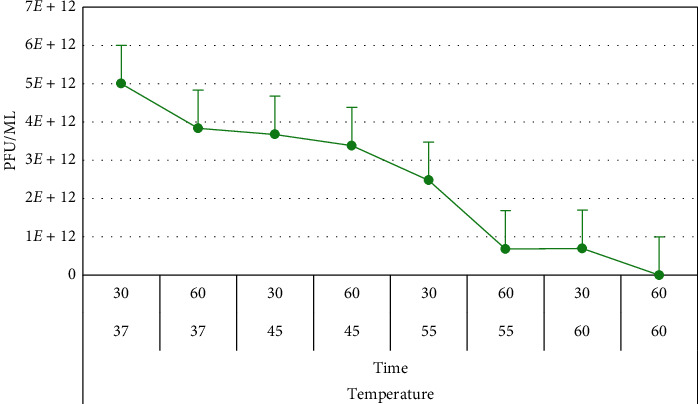
Stability of phage vB_VpM_SA3V at different temperatures.

**Figure 11:**
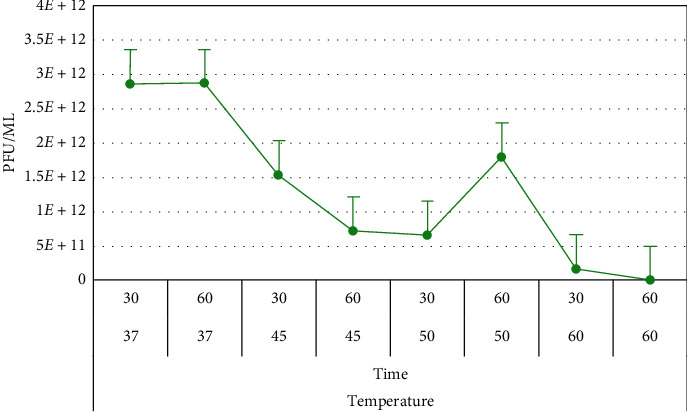
Stability of phage vB_VcM_SA3V at different temperatures.

The results showed that both phages (vB_VpM_SA3V and vB_VcM_SA3V) were stable at pH values, ranging from 4.2 to 10.0. Optimum stability for phage vB_VpM_SA3V was observed at pH 10 (Figure 8), while phage vB_VcM_SA3V displayed optimal stability at neutral pH (6-7). Both phages could not survive at the acidic pH of 3.0. The optimum temperature at which both phages were stable was 37°C, while their growth and survival rates reduced with an increase in temperature. Figures 9–11 show the stability of phages vB_VpM_SA3V and vB_VcM_SA3V to varying pH and temperatures.

## 4. Discussion

Water is an essential resource for life, and access to safe drinking water is a fundamental human need and a basic right of every individual [50]. Safe drinking water should be void of high concentrations of chemicals and minerals, as well as pathogenic microorganisms. South Africa is located in a semiarid region and receives very less rainfall, resulting in shortage of water, especially potable water. Besides, increased industrialisation and frequent establishment of informal settlements also affect the quality of water, especially in rural areas. Due to scarcity of potable water, most individuals resort to water from other unprotected sources, such as boreholes, rivers, and dams for daily activities. These unprotected sources of water could be contaminated with faecal matter from human and animal origin.

Twenty-two (38.60%) isolates were identified as *Vibrio* species through amplification of fragments of *ompW*, *rfbO1*, *flaE*, *sodB* (1), *hsp*, *sodB*, and *vhh* genes. The proportion of *V. harveyi* was higher (10 (45.45%)) compared to *V. parahaemolyticus* (5 (22.73%)), *V. mimicus* (3 (13.64%)), *V. vulnificus* (2 (9.09%)), and *V. cholerae* (2 (2.09%)). The negative isolates of these species-specific PCRs could belong to other *Vibrio* species not included in the study. Similar observations have been reported in previous studies. In these studies, some or most of these *Vibrio* species were detected in fish and shrimps [51], shrimps only [52], aquatic samples [53], marine fish, and water [54]. The *Vibrio* species identified comprised thirteen isolates from borehole water, five from tap water, and four from dam water. *Vibrio* species were dominant in samples from borehole water that do not usually undergo treatment and purification before consumption. Detection of *Vibrio* species in tap water that undergoes treatment is a cause for concern. These water sources can serve as a potential route for the transmission of *Vibrio* species to consumers and present significant public health complications when consumed. Thus, their presence in treated tap water highlights the urgent need to improve and adhere to standard operating procedures of wastewater and water purification plants.

The presence of pathogenic microorganisms associated with both self-limiting and life-threatening waterborne infections in water bodies often results from faecal pollution of human and animal origin. This is very common in rural communities and other informal settlements [55]. Poor management of wastewater treatment plants and uncontrolled sewage discharge have been identified as the two major sources of microbial pollution of water sources in South Africa [55]. There is, therefore, a need to monitor both treated water and wastewater for microbial pathogens. An assessment of adherence to standard treatment protocols and the implementation of proper management practices is, therefore, essential to improving the quality of water. Water quality assessment parameters affect optimum performance as well as the quality of the final product. These parameters include the reliability of the plant, nature of raw materials used, management of byproducts, safety, human resources, economic and financial resources, infrastructure, and maintenance [56]. These parameters are very important and should be included in the operational performance assessment tool of each water treatment plant since they directly affect the quality of the finished product. To reduce associated health risks on consumers, the limits for microbial contaminants in domestic water should fall within acceptable limits determined by the South African National Standard (SANS) 241 of 2015 [57]. Based on this, no *E. coli*, total coliform, and heterotrophic bacteria should be present in 100 mL of drinking water samples. However, *Vibrio* species is part of heterotrophic bacteria load, an indication that water does not meet the standards of SANS 241 and, therefore, considered unsafe for drinking or household use.

Three of the six virulent genes screened in this study were detected. Four out of 5 (80%) *V. parahaemolyticus* isolates, representing 18.2% of the total *Vibrios* in the study, harboured *tdh* and *trh* virulence genes. This is quite high compared to other studies with lower detection rates [53, 58] or the absence of these pathogenicity factors [52]. Nonetheless, detection rates were still lower compared to a study, whereby 31 out of 32 (96.8%) *V. parahaemolyticus* isolates from seafood possessed, at least, one (*n* = 19) or both (*n* = 13) virulent genes [10]. Three *Vibrio cholerae* isolates and 1 *Vibrio mimicus* isolate harboured the *zot* gene (18.18%). However, these results do not concur with those reported in a previous study [59]. Since the isolates did not harbour any *zot* virulent genes, contrary to the findings of this study, isolates belonging to the Harveyi clade, comprising *V. harveyi* and *V. campbellii,* possessed both typical and atypical virulent genes *vhh*, *chiA*, *vhpA*, *ToxR* (Vh), and *lux*R and serine protease. Thus, it is suggested that they might have acquired these virulence determinants from other *Vibrio* species through horizontal gene transfer [60]. There is need for constant monitoring, even in species that were previously considered to be nonpathogenic.

A large percentage (40.91% to 95.45%) of *Vibrio* species obtained in this study were resistant to streptomycin, ampicillin, and cephalothin, however, with higher sensitivity to trimethoprim-sulfamethoxazole, trimethoprim, chloramphenicol, gentamycin, tetracycline, and Ciprofloxacin. However, the percentage of multidrug resistance strains was high (72.7%). The proportion of resistant *Vibrios* to chloramphenicol, gentamicin, and tetracycline was similar to that recorded in *Vibrio* isolates from aquaculture environments. However, multidrug resistance was higher in our study than reported in these studies [61, 62].

Drug resistance is currently an issue of severe public health concern worldwide, mainly due to the alarming rate of dissemination of resistant determinants in environmental bacterial pathogens. These resistant strains have a negative impact on antibiotic therapy, resulting in difficulties in controlling and managing diseases [63, 64]. Improper discharge of municipal and industrial wastewater, as well as water from aquaculture systems, has been reported to play significant roles in the dissemination of resistant genes within aquatic ecosystems. It is worthy to note that several clinically used antibiotics are released in an active biological form through faeces and urine to the environment [65–67]. Improperly treated wastewater containing such residues increases the number of antibiotic-resistant bacteria in the environment, which could be transmitted to consumers [68–70]. Cluster analysis of bacterial growth inhibition zone diameter data also revealed very close similarities among isolates from different locations and sources, indicating similar histories of exposure to antibiotics. These findings are similar to a previous report in which cluster analysis of antibiotic-resistant data for *Escherichia coli* O157 strains revealed a closer relationship between isolates from pig and human faeces compared to cattle and humans [71].

Biofilms are an assemblage of microbial cells enclosed in a polysaccharide matrix irreversibly associated with a surface [72]. In this study, some of the *Vibrio* strains from water samples displayed the ability to bind to surfaces and form biofilms. Despite the fact that there was variability in the potential to form biofilms at different temperatures, their biofilm-forming ability presents a significant public health concern. This is because biofilms provide cells with enhanced opportunity to resist antimicrobial agents, thus persist in tissues during associated infections [73]. The ever-increasing occurrence of multidrug-resistant pathogenic bacteria poses a need for alternative sources of therapy, one of which is the use of bacteriophages [74]. Two bacteriophages (phages A and B) isolated in this study were capable of lysing many antibiotic-resistant *Vibrio* strains. These phages, therefore, provide alternative strategies for controlling these bacterial contaminants in water, given the fact that most bacterial contaminants exhibit high levels of resistance to conventional water purification chemicals. However, appropriate screening of the phage genomes for the presence of undesirable determinants that can render them unsafe is necessary [75]. The TEM images of both phages revealed they belong to the family Myoviridae based on its conserved and highly characteristic morphology. Bacteriophages belonging to this family are found everywhere in the biosphere; they reside in places such as top soils, plants, animals, or water bodies [76]. However, phages are known to be highly specific at species and strain levels, and determination of their bacterial host range is critical in determining their potential to be utilised as antimicrobial agents. Against this background, it is suggested that the virulence potential of these phages be assessed against other related bacterial species that can occur as microbial contaminants in water.

## 5. Conclusion

The findings of this study revealed the presence of antibiotic-resistant virulent *Vibrio* species from tap water, boreholes, and dams, making it unsafe for domestic use. Cluster analysis of bacterial growth inhibition zone diameter data revealed very close similarities among isolates from different sources and locations, indicating similar histories of exposure to antibiotics, which is a public health concern. Some of these *Vibrios* are capable of surviving in a wide range of ecological niches as they can form biofilms at different temperatures. Infection with such strains can persist when associated with humans. However, two phages (A and B) isolated in this study present potential characteristics as reliable and effective biocontrol agents for these strains. The phages belong to the Myoviridae family and are stable over a wide range of pH and temperatures. However, an assessment of the bacterial host range can greatly improve their chances of being used in commercial processes, and appropriate screening of the phage genomes for the presence of undesirable determinants that can render them unsafe is necessary.

## Figures and Tables

**Table 1:** Oligonucleotide primers used for PCR amplification of *Vibrio* species-specific gene fragments.

Target organism	Primer sequence (5ʹ-3ʹ)	Targeted gene	Amplicon size (bp)	PCR cycling conditions	Reference
All bacterial strains (universal 16S rRNA gene sequence)	27F: AGAGTTTGATCATGGCTCAG 1492R: GGTACCTTGTTACGACTT	16S rRNA	1420	Initial denaturation at 94°C for 3 minutes, 25 cycles of denaturation at 94°C for 1 minute, primer annealing at 55°C for 1 minute, elongation at 72°C for 2 minutes, and a final strand elongation step at 72°C for 10 minutes.	[37]

*V. cholerae*	F: CACCAAGAAGGTGACTTTATTGTG R: GGTTTGTCGAATTAGCTTCACC	*ompW*	304	Initial denaturation at 94°C for 10 minutes, 30 cycles of denaturation at 94°C for 1 minute, primer annealing at 59°C for 1 minute, elongation at 72°C for 2 minutes, and a final strand elongation at 72°C for 10 minutes.	[38]
*V. cholerae* serogroup O1	F: TCTATGTGCTGCGATTGGTG R: CCCCGAAAACCTAATGTGAG	*rfbO1*	638		

*V. cholerae*	F: AAGACCTCAACTGGCGGTA R: GAAGTGTTAGTGATCGCCAGAGT	*sodB* (1)	248	Initial denaturation at 95°C for 10 minutes, 35 cycles of denaturation at 92°C for 40 seconds, primer annealing at 57°C for 1 minute, elongation at 72°C for 1.5 minutes, and a final strand elongation at 72°C for 10 minutes.	[39]
*V. parahaemolyticus*	F: GCAGCTGATCAAAACGTTGAGT R: ATTATCGATCGTGCCACTCAC	*flaE*	897		
*V. vulnificus*	F: GTCTTAAAGCGGTTGCTGC R: CGCTTCAAGTGCTGGTAGAAG	*hsp60*	410		
*V. mimicus*	F: CATTCGGTTCTTTCGCTGAT R:GAAGTGTTAGTGATTGCTAGAGAT	*sodB*	121		

*V. harveyi*	F: CTTCACGCTTGATGGCTACTG R: GTCACCCAATGCTACGACCT	*vhh*	235	Initial denaturation at 95°C for 10 minutes, 30 cycles of denaturation at 95°C for 1 minute, primer annealing at 50°C for 1 minute, elongation at 72°C for 1 minute, and a final strand elongation at 72°C for 10 minutes.	[40]

**Table 2:** Primer sequences and PCR amplification conditions for the detection of *Vibrio* virulent genes.

Target gene	Primer sequence (5ʹ-3ʹ)	Amplicon size (bp)	PCR cycling conditions	Reference
*tdh*	F: GTAAAGGTCTCTGACTTTTGGAC R: TGGAATAGAACCTTCATCTTCACC	270	Initial denaturation at 94°C for 3 minutes, 30 cycles of denaturation at 94°C for 1 minute, primer annealing at 58°C for 1 minute, elongation at 72°C for 1 minute, and a final strand elongation at 72°C for 5 minutes.	[10]
*trh*	F: TTGGCTTCGATATTTTCAGTATCT R: CATAACAAACATATGCCCATTTCCG	486		

*ctxAB*	F: GCCGGGTTGTGGGAATGCTCCAAG R: GCCATACTAATTGCGGCAATCGCATG	536	Initial denaturation at 94°C for 10 minutes, 30 cycles of denaturation at 94°C for 1 minute, primer annealing at 59°C for 1 minute, elongation at 72°C for 2 minutes, and a final strand elongation at 72°C for 10 minutes.	[41]
*zot*	F: TCGCTTAACGATGGCGCGTTTT R: AACCCCGTTTCACTTCTACCCA	947		

*flrA*	F: GAGGCAACAGCACCATCAAA R: CGCATCTATATCAGGGACAA	503	Initial denaturation at 95°C for 5 minutes, 35 cycles of denaturation at 95°C for 45 seconds, primer annealing at 72°C for 45 seconds, and a final strand elongation at 72°C for 15 minutes.	[8]
*vpsR*	F: GGTGAGTAGCCATAAGCAAG R: CATCCAGCACCACAGTATCT	911		

**Table 3:** Distribution patterns of *Vibrio* species from different sampling types and sites.

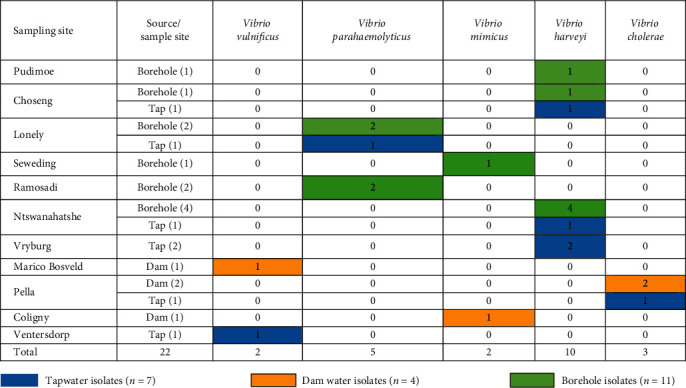

**Table 4:** Number and percentage of antibiotic resistance of *Vibrio* isolates recovered from water samples.

Antibiotic (disc content)	Resistant (%)	Intermediate (%)	Susceptible (%)
Ampicillin (10 *μ*g)	17 (77.3)	2 (9.1)	3 (13.6)
Cephalothin (30 *μ*g)	21 (95.5)	1 (4.5)	0 (0.0)
Chloramphenicol (30 *μ*g)	2 (9.1)	2 (9.1)	18 (81.8)
Ciprofloxacin (5 *μ*g)	1 (4.5)	7 (31.8)	14 (63.6)
Tetracycline (30 *μ*g)	5 (22.7)	1 (4.5)	16 (72.7)
Gentamicin (10 *μ*g)	3 (13.6)	2 (9.1)	17 (77.3)
Kanamycin (30 *μ*g)	3 (13.6)	8 (36.4)	11(50.0)
Nalidixic acid (30 *μ*g)	6 (27.3)	8 (36.4)	8 (36.4)
Trimethoprim-sulfamethoxazole (1.25/23.75 *μ*g)	1 (4.5)	1 (4.5)	20 (90.9)
Streptomycin (10 *μ*g)	9 (40.9)	7 (31.8)	6 (27.3)
Trimethoprim (5 *μ*g)	3 (9.1)	1 (4.5)	18 (81.8)

## Data Availability

The data used to support the findings of this study are included within the article.

## Supplementary Materials

Supplementary Materials Supplementary Table 1: the number of water samples collected from the different areas during the study.
