# Supplementary material for: Characterisation of Vibrio Species from Surface and Drinking Water Sources and Assessment of Biocontrol Potentials of Their Bacteriophages
Source: Int J Microbiol. 2020 Aug 4;2020:8863370. doi: 10.1155/2020/8863370 (PMC7424396; doi:10.1155/2020/8863370)
Supplement: Supplementary Materials — Supplementary Table 1: the number of water samples collected from the different areas during the study. [file 8863370.f1.docx]

**SUPPLEMENTARY TABLE 1: The number of water samples collected from the different areas during the study.**

| **Sample area** | **Water samples** | | |
| --- | --- | --- | --- |
|  | **Tap** | **Borehole** | **Dam** |
| Dryharts | 4 |  |  |
| Choseng | 4 | 4 |  |
| Pudimoe | 4 | 4 |  |
| Ntswanahatshe | 4 | 4 |  |
| Vryburg | 4 |  |  |
| Pella | 4 | 4 | 2 |
| Silverkraans | 4 |  |  |
| Swartruggens | 4 | 4 |  |
| Coligny | 4 | 4 | 2 |
| Ventersdorp | 4 |  |  |
| Lichtenburg | 4 |  |  |
| Marico Bosveld |  |  | 2 |
| Mooiriver | 4 |  |  |
| Potchefstroom | 4 |  |  |
| Phola | 4 | 6 |  |
| Ramosadi | 4 | 6 |  |
| Motlhabeng | 4 | 6 |  |
| Dibate | 4 | 6 |  |
| Seweding | 4 | 6 |  |
| Lonely park | 2 | 2 |  |
| **Total** | **74** | **56** | **6** |
